# Vitamin D in the Prevention and Treatment of Oral Cancer: A Scoping Review

**DOI:** 10.3390/nu15102346

**Published:** 2023-05-17

**Authors:** Man Hung, Konstantinia Almpani, Bao Thao, Kaili Sudweeks, Martin S. Lipsky

**Affiliations:** 1 College of Dental Medicine, Roseman University of Health Sciences, South Jordan, UT 84095, USA; 2 Department of Orthopaedics, University of Utah, Salt Lake City, UT 84108, USA; 3 Department of Educational Psychology, University of Utah, Salt Lake City, UT 84109, USA; 4 School of Business, University of Utah, Salt Lake City, UT 84112, USA; 5 Department of Veterans Affairs Medical Center, Salt Lake City, UT 84148, USA; 6 College of Health Sciences, Utah Tech University, St. George, UT 84770, USA; 7 Institute on Aging, Portland State University, Portland, OR 97201, USA

**Keywords:** oral carcinogenesis, nutrition, clinical outcomes, evidence-based medicine, evidence-based dentistry

## Abstract

*Introduction*: Oral cancer is a serious health problem with an increasing incidence worldwide. Researchers have studied the potential anti-cancerous action of vitamin D and its association with several cancers including oral cancer. The purpose of this scoping review is to synthesize the existing literature on the role of vitamin D on oral cancer. *Methods:* A scoping review of the literature was conducted using the framework developed by Arkey and O’Malley and the PRISMA-ScR guidelines. Nine databases were searched for peer-reviewed human studies published in English that either investigated the association of vitamin D with, or its impact on either the prevention or treatment of oral cancer. The authors then extracted data using a predefined form to summarize information about article type, study design, participant characteristics, interventions, and outcomes. *Results*: Fifteen articles met the review criteria. Among the 15 studies, 11 were case–control, 3 were cohort studies, and 1 was a clinical trial. In four studies, the evidence supported a preventive action of vitamin D against oral cancer and a reduction in the negative side effects associated with chemo- and radiotherapy. Several studies that focused on genetic polymorphisms and the expression of the 1,25 dihydroxyvitamin D3 receptor (VDR) suggested significant associations with vitamin D and increased oral cancer risk and worse survival rates. In contrast, two studies did not reveal a strong association between vitamin D and oral cancer. *Conclusions*: The current evidence suggests an association between vitamin D deficiency and an increased risk of oral cancer. VDR gene polymorphisms might also be a part of future preventive and therapeutic strategies against oral cancer. Carefully designed studies are required to explore and define what role, if any, vitamin D might play in the prevention and treatment of oral cancer.

## 1. Introduction

Oral cancer accounts for about 3% of all cancers in the United States [1] and includes cancers of the lip, tongue, salivary glands, and other sites in the mouth. It is the 16th most common type of cancer worldwide [2]; According to the American Cancer Society, an estimated 54,000 people in the United States will develop oral cancer annually and over 11,000 individuals will die from it [3]. Unfortunately, the incidence of oral cancer is likely to increase in the future [4].

Risks associated with oral cancer include male sex, genetic and epigenetic factors, tobacco use, alcohol use, betel quid chewing, human papillomavirus infection (HPV), bacterial infections, and immunosuppressive agents [5,6,7,8,9]. Often first diagnosed by dentists, treatment options for oral cancer depend on the stage and tumor type and can include surgery, radiotherapy, chemotherapy, and gene therapy [10,11]. The clinical recognition and evaluation of oral mucosal lesions can detect up to 99% of oral cancers and premalignant lesions, and early detection remains the most important determinant of the treatment outcome in oral cancer [12]. Despite opportunities for early intervention, the disease burden for oral cancer continues to be substantial.

One potential option to reduce the morbidity and mortality of oral cancer is vitamin D. Vitamin D is an essential fat-soluble vitamin that regulates calcium and phosphorus, elements critical to healthy bones and teeth. It can be obtained from dietary supplements, food, or made by skin exposed to sunlight. In addition to calcium and phosphorus homeostasis, vitamin D promotes cell growth, helps to regulate inflammation, prostaglandin synthesis and apoptosis, and inhibits metastasis through a variety of mechanisms that affect growth factors [13,14]. Preclinical studies strongly support the cancer prevention properties of vitamin D because of its pro-apoptotic, anti-proliferative, and anti-angiogenic performances against a wide range of cancer cells [15].

In addition to credible physiologic mechanisms supporting the hypothesis that vitamin D improves cancer outcomes, epidemiologic studies also demonstrate a link between vitamin D deficiency and cancer [16,17]. Colorectal, breast, leukemia, prostate, and pancreatic cancers are the cancers for which the most human data are available [18,19,20,21]. However, fewer studies explore whether there is a link between oral cancer and vitamin D. Aspects of OSCC, such as the oral cavity’s unique environment and its association with alcohol, tobacco, and HPV, might make the impact of Vitamin D on oral cancer differ from its effect on other cancers. Given the morbidity and mortality of oral cancer, identifying effective preventive and treatment strategies is important.

The goal of this scoping review is to provide an overview of the evidence regarding the association between vitamin D and the prevention/treatment of oral cancer. This review should broaden the scientific understanding of what role, if any, vitamin D may play in the prevention and treatment of oral cancer, as well as identifying gaps in the research regarding this function of vitamin D.

## 2. Methods

### 2.1. Protocol and Eligibility Criteria

This review used the scoping review framework developed by Arksey and O’Malley [22] and followed the guidelines of the PRISMA Extension for Scoping Reviews (PRISMA-ScR) [23]. In contrast to systematic reviews, scoping reviews are useful for answering broader questions about a topic. While they follow a systematic approach to map evidence about a topic, they represent a more preliminary assessment of the literature and typically use broader criteria and less rigorous study selection and data extraction [24,25]. Eligible articles included peer-reviewed human studies published in English that either investigated the association or impact of vitamin D and either the prevention or treatment of oral cancer. Searches were conducted up to 30 June 2022, with no year restriction for when the search began. Table 1 provides a more detailed outline of the inclusion/exclusion criteria for article selection.

### 2.2. Information Sources and Search

To ensure a comprehensive search, the searched databases included Medline, Web of Science, Science Direct, Scopus, Google Scholar, Cochrane Library, ProQuest, and the Ovid database. Table 2 outlines specific search strategies and keywords for each database. One author (K.S.) reviewed the references for each article included in the review to identify other potentially relevant studies for inclusion.

### 2.3. Selection of Sources of Evidence and Data Charting Process

One author (K.S.) screened the titles and abstracts of those articles identified after applying the search terms to the databases to assess their relevance to the scoping review topics; articles that did not contain content related to vitamin D and oral cancer prevention and treatment were excluded. A second author (K.A.) assessed any articles that could not be designated for inclusion or exclusion by the initial screening. The two authors (K.S. and K.A.) then met and discussed these until they achieved agreement on inclusion or exclusion. If eligibility could not be decided by title or abstract, the full text of the article was retrieved and assessed to determine eligibility. Study selection was performed unmasked (i.e., knowing the authors’ identities) since scientific evidence does not strongly recommend masked assessment [26]. Two authors (B.T. and K.S.) then independently reviewed the relevant articles using a predefined data extraction form to summarize information about the article type, study design, participant characteristics, interventions, and outcomes, and whether the study was related to prevention and/or treatment. Any differences between the two reviewers’ (B.T. and K.S.) data extraction forms were resolved after consultation with a third author (K.A.) and the three reviewers (B.T., K.S. and K.A.) achieved consensus. Author M.H. audited quality, the sources of evidence, and data charting to confirm the validity of the results.

### 2.4. Data Items and Synthesis of the Results

The thematic categories of this scoping review were articles containing research related to vitamin D and the treatment and prevention of oral cancer. The results section uses these two categories to report and synthesize results.

## 3. Results

### Selection, Characteristics of Sources of Evidence, and Summary Results

**Figure 1:**
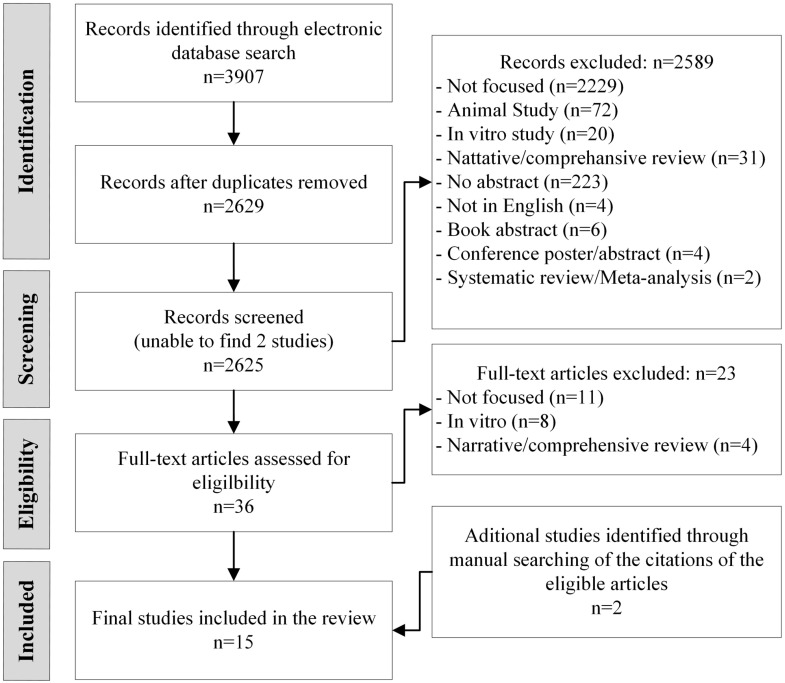
PRISMA-ScR study selection.

The initial search identified 3907 articles and, after eliminating duplicate articles and articles that could not be retrieved, 2627 remained. After reviewing titles and abstracts, 36 full-text articles were reviewed and 15 met the review criteria (Figure 1). Among the 15 studies, 11 were case–control, 3 were cohort studies and 1 was a clinical trial. Table 3 provides a detailed description of the articles and summarizes the results of the individual sources of evidence.

Publication dates ranged from 2000 to 2022. Of the 15 studies, 6 studies explored genetic polymorphisms and the expression of the 1,25 dihydroxyvitamin D3 receptor (VDR), which mediates the pleiotropic biological actions of vitamin D [27,29,32,33,35,40]. A significant association was detected between the VDR Taq I heterozygous Tt genotype and an increased risk of oral squamous cell carcinoma (OSCC) [29]. Investigations of Taq I also found that VDR tt may have a protective effect from OSCC for females [29]. One study investigated the links between oral cancer and VDR gene polymorphisms (CYP27B1 and CYP24A1), which regulate the anabolism and catabolism of vitamin D, and found that the CYP24A1 gene polymorphism might influence oral cancer and that the VDR FokI polymorphism was associated with worse survival and could be an independent prognostic marker [42]. In a genetic study describing the results of two different single nucleotide VDR polymorphisms (SNPs rs2107301 and rs2238135), a statistically significantly higher frequency of the SNP rs2238135 G/C genotype occurred in the oral cavity cancer group [35].

In contrast, other studies found that VDR expression was not associated with clinicopathological characteristics of OSCC. However, decreased VDR expression in OSCC was associated with tumor relapse, suggesting that vitamin D could be an effective adjuvant chemoprevention agent for residual tumor cells [32]. In a later study, Grimm et al. found that VDR expression was reduced in precancerous lesions and that either natural or synthetic vitamin D compounds induced the apoptosis of VDR+ cells in oral precancerous and OSCC lesions [33]. These findings suggest that vitamin D might be an effective chemoprevention treatment strategy.

Grimm et. al. [33] also found that vitamin D deficiency increased the risk of developing OSCC from OPMDs, while optimal vitamin D levels increased the antitumor immune response and were associated with a lower risk of OSCC. They also concluded that tobacco could increase the risk of developing OSCC by altering vitamin D levels and that vitamin D supplements decrease the adverse effects associated with treatment, such as mucositis and pain. Another study examined the effect of vitamin D supplementation on quality of life (QOL) and oral cancer and concluded that vitamin D significantly reduced therapy-related toxicities, reduced morbidities, and improved QOL parameters [27]. In the same study, vitamin D levels were significantly lower in oral cancer cases compared to a healthy control group.

A clinical trial comparing baseline vitamin D levels in healthy and OSCC cases revealed that severe vitamin D deficiency was associated with an increased risk of oral cancer [38]. A second study similarly identified a statistically significant inverse association between healthy vitamin D levels and digestive tract tumors and oral cancer in men [31], but not in other tumors such as brain, melanoma, multiple myeloma, and lung. In contrast, a Finnish study did not find an association between low serum 25(OH)D and the risk of head and neck cancer [28].

In a clinical trial, patients with oral cancer lesions exhibited lower levels of inflammatory mediators, such as cytokines and adipokines, than healthy controls; it was also found that vitamin D supplements improved those levels [39]. The role of vitamin D and markers of oxidative stress and/or inflammation was also explored, with lower levels of vitamin D and disturbed oxidant-antioxidant homeostasis detected in oral cancer subjects [37].

In an older case–control study, vitamin D was associated with a reduced oral cancer risk, but the association was not as strong as the association for other micronutrients [36]. Finally, according to the results of a mendelian randomization study, which helps account for several potential confounding variables, no strong evidence of a causal association was identified with oral or oropharyngeal cancer [30]. The effect of vitamin D on oral cancer progression was not assessed in this study.

## 4. Discussion

### 4.1. Summary of Evidence

Most studies that examined vitamin D levels in oral cancer patients compared to healthy controls found that vitamin D deficiency was positively associated with the incidence of oral cancer [27,28,31,33,34,37,38]. Three studies supported the concept of maintaining healthy levels of vitamin D as part of a preventive strategy for oral cancer [28,31,34]. One study provided evidence that including vitamin D in treatment regimens might mitigate side effects associated with the chemo- and radiotherapy of oral cancerous lesions and improve the QOL of the patients [27]). A systematic review by Maturana-Ramirez also found an association of hypovitaminoses D with lower survival rates in patients with OSCC, a greater incidence of post-operative recurrence, and an increase in adverse reactions to chemotherapy [43]. Given the low cost and low toxicity of vitamin D, vitamin D supplementation represents an attractive option that merits further exploration.

However, while more studies correlated low vitamin D levels with oral cancer, in a case–control study that examined the association of certain micronutrients and oral cancer, Negri et al. concluded that vitamin D was only weakly associated with oral cancer [36]. In addition, based on the analysis of unconfounded genetic data from three large-scale studies, Dudding et al. concluded that it was unlikely that vitamin D significantly lowers oral cancer risk [30]. While there is a theoretical basis for using Vitamin D as a preventive agent, our review did not find a high-quality randomized controlled trial investigating the efficacy of vitamin D supplementation for OSCC chemoprevention. Low vitamin D levels are associated with a higher risk of other chronic health conditions, such as cardiovascular disease, diabetes, and autoimmune disorders [44]. The restoration of vitamin D deficits induced by ill health could explain why low-dose supplementation led to slight gains in survival for oral cancer in some studies, rather than a cancer-related chemoprotective effect [45]. Since the evidence is mixed, our review suggests that additional research, such as a large prospective clinical trial with high-risk groups or individuals with premalignant lesions, might help evaluate vitamin D’s potential as a chemoprotective agent.

Less evidence exists regarding the use of vitamin D as part of a treatment strategy to improve oral cancer outcomes. Based on one study, vitamin D supplementation helped normalize inflammatory modulators associated with oral cancer, suggesting a potential therapeutic effect [39].

Several studies investigated VDR gene polymorphisms and expression. The results of this research suggest a role in the identification of potential gene therapy targets and vitamin D [27,29,32,33,40]. Similarly, a meta-analysis of tobacco-related cancers, including OSCC, found a correlation between the TaqI polymorphism of VDR and the risk of tobacco-related cancers [46]. Both tobacco and alcohol increase the risk of oral cancer [30], and one contributing factor may be that tobacco use alters vitamin D levels [43,47]. Moreover, excessive alcohol consumption has been associated with VDR CYP27B1 polymorphism, which is correlated with an increased risk for oral cancer [40]. Although little current clinical evidence supports using vitamin D as a treatment modality that lowers mortality, this review suggests that the existing evidence merits an exploration of the effect of vitamin D supplementation on OSCC outcomes and whether it helps both those with low and normal levels. Data linking vitamin D and oral cancer with alcohol, tobacco use, and gender also highlight the importance of subgroup analysis.

### 4.2. Limitations

Despite the extensive literature search that incorporated multiple databases, the number of studies with a controlled methodology was limited and only one clinical trial could be found for the review. In addition, variability among the populations studied, such as differences in the ethnicity, nationality, and gender of the subjects, make it difficult to gauge whether these differences might account for discrepancies in results between studies. Furthermore, the definition of vitamin D deficiency was not always consistent across the studies examined. For example, one study estimated 25(OH)D levels and categorized them into five levels (sufficient, insufficient, mildly deficient, moderately deficient, and severely deficient [27]), while another study considered vitamin D deficiency for oral cancer patients as having statistically significant lower concentration of 25(OH)D than the healthy group [37]. Such differences in the definition of vitamin D deficiency might affect the interpretation of the results.

## 5. Conclusions

The currently available scientific evidence suggests a possible association between vitamin D deficiency and an increased risk of oral cancer. Some evidence also supports the hypothesis that vitamin D may be useful in oral cancer prevention strategies. Carefully designed studies are required to explore and define what role, if any, vitamin D may play in the prevention and treatment of oral cancer.

Finally, specific polymorphisms of the VDR gene have been directly linked to oral cancer and could represent a pathway for developing new preventive and therapeutic interventions. More clinical trials and genetic studies that include ethnically diverse populations are required to define the possible role of vitamin D in the prevention and treatment of oral cancer.

## Figures and Tables

**Table 1:** Inclusion and exclusion criteria for article selection.

Criteria Category	Inclusion Criteria	Exclusion Criteria
Outcome	- Studies investigating the association between Vitamin D and oral cancer	- Investigations not relevant to the subject of this study
Study design	- Randomized controlled clinical trials - Prospective clinical trials - Retrospective clinical trials - Case–control observational studies - Cross-ectional surveys - Case series - Case reports	- Narrative reviews - Unsupported opinions of experts - Editors’ choices - Replies to the author/editor - Book abstracts - Conference abstracts - Ongoing studies - Animal studies - In vitro studies - In silico studies - Meta-analyses - Systematic reviews - Studies with missing English abstract or no abstract at all
Participant characteristics	- Studies included should involve only human subjects of any age and gender.	- Investigations in patients with syndromes or genetic disorders

**Table 2:** The electronic databases, search strategies, and corresponding number of articles.

Electronic Databases	Search Strategies	Extent of Search	Number of Articles
Medline www.ncbi.nlm.nih.gov/sites/entrez (Accessed on 20 July 2022)	((Vitamin D) OR (Vitamin D2) OR (Vitamin D3) OR (Calcidiol) OR (Calcitriol) OR (Ergocalciferol) OR (Cholecalciferol) OR (25-hydroxyvitamin D) OR (25(OH)D) OR (Vit D) OR (Vit D2) OR (Vit D3)) AND ((Oral Cancer*) OR (Mouth Cancer*) OR (Oral Neoplasm*) OR (Mouth Neoplasm*) OR (Oral Carcinoma*) OR (Mouth Carcinoma*) OR (Oral Malignancy) OR (Oral malignancies) OR (Mouth Malignancy) OR (Mouth Malignancies) OR (Oral Tumor*) OR (Mouth Tumor*))	In all fields	1035
Web of Science https://clarivate.com/webofsciencegroup/solutions/web-of-science/ (Accessed on 20 July 2022)	(((((((((((ALL=(Vitamin D)) OR ALL=(Vitamin D2)) OR ALL=(Vitamin D3)) OR ALL=(Vit D)) OR ALL=(Vit D2)) OR ALL=(Vit D3)) OR ALL=(Calcitriol)) OR ALL=(Calcidiol)) OR ALL=(Ergocalciferol)) OR ALL=(Cholecalciferol)) OR ALL=(25-hydroxyvitamin)) OR ALL=(25(OH)D) AND (((((((((((ALL=(Oral Cancer*)) OR ALL=(Mouth cancer*)) OR ALL=(Oral Neoplasm*)) OR ALL=(Mouth Neoplasm*)) OR ALL=(Oral Carcinoma*)) OR ALL=(Mouth Carcinoma*)) OR ALL=(Mouth Malignancy)) OR ALL=(Mouth Malignancies)) OR ALL=(Oral Malignancy)) OR ALL=(Oral malignancies)) OR ALL=(Oral Tumor*)) OR ALL=(Mouth Tumor*)	In all fields	1532
Science Direct http://www.sciencedirect.com (Accessed on 20 July 2022)	(“Vitamin D” OR “Vitamin D2” OR “Vitamin D3”) AND (“Oral Cancer” OR “Oral Neoplasm” OR “Oral Carcinoma” OR “Oral Malignancy” OR “Oral Tumor”)	Search filtered by article type inclusion/exclusion criteria	332
Wiley Online Library http://onlinelibrary.wiley.com (Accessed on 20 July 2022)	(“Vitamin D” OR “Vitamin D2” OR “Vitamin D3” OR “Calcidiol” OR “Calcitriol” OR “Ergocalciferol” OR “Cholecalciferol” OR “25-hydroxyvitamin D” OR “25(OH)D” OR “Vit D” OR “Vit D2” OR “Vit D3”) AND (“Oral Cancer*” OR “Mouth Cancer*” OR “Oral Neoplasm*” OR “Mouth Neoplasm*” OR “Oral Carcinoma*” OR “Mouth Carcinoma*” OR “Oral Malignancy” OR “Oral malignancies” OR “Mouth Malignancy” OR “Mouth Malignancies” OR “Oral Tumor*” OR “Mouth Tumor*”)	Anywhere	421
Scopus www.scopus.com (Accessed on 20 July 2022)	((Adults) OR {Vitamin D2} OR {Vitamin D3} OR {Calcidiol} OR {Calcitriol} OR {Ergocalciferol} OR {Cholecalciferol} OR {25-hydroxyvitamin D} OR {25(OH)D} OR {Vit D} OR {Vit D2} OR {Vit D3}) AND ({Oral Cancer*} OR {Mouth Cancer*} OR {Oral Neoplasm*} OR {Mouth Neoplasm*} OR {Oral Carcinoma*} OR {Mouth Carcinoma*} OR {Oral Malignancy} OR {Oral malignancies} OR {Mouth Malignancy} OR {Mouth Malignancies} OR {Oral Tumor*} OR {Mouth Tumor*})	No limits	28
Google Scholar www.scholar.google.com (Accessed on 20 July 2022)	((Vitamin D) OR (Vitamin D2) OR (Vitamin D3) OR (Calcidiol) OR (Calcitriol) OR (Ergocalciferol) OR (Cholecalciferol) OR (25-hydroxyvitamin D) OR (25(OH)D) OR (Vit D) OR (Vit D2) OR (Vit D3)) AND ((Oral Cancer*) OR (Mouth Cancer*) OR (Oral Neoplasm*) OR (Mouth Neoplasm*) OR (Oral Carcinoma*) OR (Mouth Carcinoma*) OR (Oral Malignancy) OR (Oral malignancies) OR (Mouth Malignancy) OR (Mouth Malignancies) OR (Oral Tumor*) OR (Mouth Tumor*))	All fields	13,300—results from first 40 pages were included = 405
Ovid database http://ovidsp.ovid.com/autologin.html (Accessed on 20 July 2022)	((Vitamin D) OR (Vitamin D2) OR (Vitamin D3) OR (Calcidiol) OR (Calcitriol) OR (Ergocalciferol) OR (Cholecalciferol) OR (25-hydroxyvitamin D) OR (Vit D) OR (Vit D2) OR (Vit D3)) AND ((Oral Cancer*) OR (Mouth Cancer*) OR (Oral Neoplasm*) OR (Mouth Neoplasm*) OR (Oral Carcinoma*) OR (Mouth Carcinoma*) OR (Oral Malignancy) OR (Oral malignancies) OR (Mouth Malignancy) OR (Mouth Malignancies) OR (Oral Tumor*) OR (Mouth Tumor*))	All fields	120
ProQuest http://search.proquest.com/ (Accessed on 20 July 2022)	(((Vitamin D) OR (Vitamin D2) OR (Vitamin D3) OR (Calcidiol) OR (Calcitriol) OR (Ergocalciferol) OR (Cholecalciferol) OR (25-hydroxyvitamin D)) AND ((Oral Cancer) OR (Mouth Cancer) OR (Oral Neoplasm) OR (Mouth Neoplasm) OR (Oral Carcinoma) OR (Mouth Carcinoma) OR (Oral Malignancy) OR (Oral Tumor) OR (Mouth Tumor)))	-Abstract -English language	31
Cochrane Library http://onlinelibrary.wiley.com/cochranelibrary/ (Accessed on 20 July 2022)	“Vitamin D” OR “Vitamin D2” OR “Vitamin D3” OR “Calcidiol” OR “Calcitriol” OR “Ergocalciferol” OR “Cholecalciferol” OR “25-hydroxyvitamin D” OR “25(OH)D” OR “Vit D” OR “Vit D2” OR “Vit D3” AND “Oral Cancer*” OR “Mouth Cancer*” OR “Oral Neoplasm*” OR “Mouth Neoplasm*” OR “Oral Carcinoma*” OR “Mouth Carcinoma*” OR “Oral Malignancy” OR “Oral malignancies” OR “Mouth Malignancy” OR “Mouth Malignancies” OR “Oral Tumor*” OR “Mouth Tumor*”	No limits	2

**Table 3:** Characteristics and result summaries of the studies included in the scoping review.

Author (Year)	Country	Study Type/Design	Study Population	Results Summary	Category
Anand at el. (2017) [27]	India	Prospective/Cohort	Total cases: N = 87; 69 male, 18 female; mean age of 43 (32–53) * years	Vitamin D supplementation reduced treatment-related toxicities.	Treatment
			Controls: N = 95; matched gender; median age of 49 (35–62) years		
Arem et al. (2011) [28]	Finland	Prospective/Case–control	Total cases: N = 182 male; median age of 57 (53–61) * years	Did not show an association between serum 25(OH)D and risk of head and neck cancers.	Prevention
			Controls: N = 182 male; 57 (53–61) *		
Bektas-Kayhan et al. (2010) [29]	Turkey	Prospective/Case–control	Total cases: N = 64; 43 male, 21 female; median age of 55 (42–69) years	VDR Tt genotype was linked to an increased risk of OSCC.	Prevention
			Controls: N = 87; 45 male, 42 female; median age of 57 (44–70) years	VDR gene influences susceptibility to OSCC.	
Dudding et al. (2018) [30]	United Kingdom	Retrospective/Cohort	Total cases: N = 5133; 3798 male, 1335 female; age N/A	Found no clinically relevant protective effect of 25OHD on oral cancers; supplementation unlikely to be beneficial/	Prevention
			Control: N = 5984; 3882 male, 2102 female; age N/A		
Giovannucci et al. (2006) [31]	United States	Prospective/Case–control	Total cases: N = 51 males; aged 40–75 years	Investigated the association of multiple determinants of vitamin D exposure to cancer risk and found a statistically significant inverse association.	Prevention
			Controls: N/A		
Grimm et al. (2013) [32]	Germany	Retrospective/Case–control	Total cases: N = 191; 145 male, 46 female; ~60 years of age	Low VDR expression is associated with tumor recurrence, and low VDR expression is an independent prognostic factor.	Prevention, Treatment
			Controls: N = 10; gender N/A; age N/A		
Grimm et al. (2015) [33]	Germany	Retrospective/Case–control	Total cases: N = 42; 24 male, 18 female; age N/A	Serum vitamin D levels correlated with apoptosis induction of VDR+ cells in oral precancerous lesions; OSCC by natural or synthetic vitamin D compounds could be useful for chemoprevention.	Prevention
			Controls: N = 5; gender N/A; age N/A		
Lipworth et al. (2009) [34]	Italy	Prospective/Case–control	Total cases: N = 804 cases; 658 male, 146 female; median age of 58 (22–78) years	Observed inverse associations between dietary vitamin D intake and risk and oral cancer, which were most pronounced among current heavy smokers.	Prevention
			Controls: N = 743; 593 male, 150 female; median age of 60 (36–77) years		
Małodobra-Mazur et al. (2012) [35]	Poland	Prospective/Cohort	Total cases: N = 73; 48 male, 25 female; median age of 58 (27–83) years	Provided evidence for the genetic association between the specific VDR gene polymorphism and the occurrence and risk of oral cavity cancer.	Prevention
			Controls: N = 100 male; age N/A		
Negri et al. (2000) [36]	Italy	Retrospective/Case–control	Total cases: N = 344; 274 male, 70 female with oral cancer and N = 410; 364 male, 46 female, with pharyngeal cancer; median age of 57 (22–77) years	Dietary vitamin D was inversely associated with oral cancer risk.	Prevention
			Controls: N = 1.775; 1.254 male, 521 female; median age of 57 (20–78) years		
Nuszkiewicz et al. (2021) [37]	Poland	Prospective/Case–control	Total cases: N = 45; 29 male, 16 female; median age 57–71 years	Vitamin D deficiency and disturbed oxidant–antioxidant homeostasis are more common in oral cancer patients.	Prevention
			Controls: N = 25; 11 male, 14 female; median age 55 (54–57) years		
Udeabor et al. (2020) [38]	Saudi Arabia	Prospective/Case–control	Total cases: N = 51; 22 male, 29 female; median 59 (46–72) years	Found an association between vitamin D deficiency and OSCC risk, especially in levels below 25 ng/mL	Prevention
			Controls: N = 113; 36 male, 77 female; median age of 49 (33–65) years		
Young et al. (2015) [39]	United States	Prospective/Clinical trial	Total cases: N = 61; 40 male, 21 female; age range 46–72 years	Treatment with vitamin D in patients with head and neck cancers helped maintain normal immune reactivity.	Treatment
			Controls: N = 30; 19 male, 11 female; mean age of 59 (48–70) years		
Zeljic et al. (2012) [40]	Serbia	Prospective/Case–control	Total cases: N = 110; 81 male, 29 female; mean age of ~60 years	Found a significant decrease in oral cancer risk in VDR heterozygote AG of CYP24A1 gene compared with wild-type AA genotype.	Prevention
			Controls (matched): N = 122	VDR FokI polymorphism was associated with decreased survival and could be an independent prognostic marker.	
Zhang et al. (2015) [41]	China	Prospective/Case–control	Total cases: N = 70; 62 male, 8 female; median age 51 (40–63) years	Serum vitamin D was not significantly different between the OSCC cases and the controls.	Prevention
			Controls: N = 70; 62 male, 8 female; median age 53 (42–64) years		

Note: P = pre-malignant, OSCC = oral squamous cell carcinoma, HNSCC = head and neck squamous cell carcinoma, pts = patients and VDR = Vitamin D receptor. * Number for the entire cohort, which also included cases of other cancer types. Synthesis of results.

## Data Availability

All data are available via the electronic database links and search strategies provided in Table 2 of the manuscript.
